# Remimazolam use for awake craniotomy

**DOI:** 10.1186/s40981-021-00428-w

**Published:** 2021-03-15

**Authors:** Shunsuke Tachibana, Kengo Hayamizu, Michiaki Yamakage

**Affiliations:** Department of Anesthesiology, Sapporo Medical University School of Medicine, South 1, West 16, Chuo-ku, Sapporo, Hokkaido 060-8543 Japan

To the Editor,

We read the case reports by Sato et al. [1] and Yoshida et al. [2] with great interest. Certainly, remimazolam is an ultrashort-acting drug and may be suitable for the asleep-awake-asleep technique of awake craniotomy (AC) [3]. Moreover, the use of flumazenil can antagonize the effects of remimazolam, and we believe that remimazolam is a beneficial drug for ensuring an awake state and shortening the time to extubation. We have also experienced two AC procedures in which remimazolam was used (case 1: male, 85 kg; case 2: male, 87 kg). However, at this stage, we cannot affirm that remimazolam can be safely and successfully used for anesthetic management of AC.

We are interested to hear the opinions of JACR readers regarding the following three points. First, we have the impression that the ability of remimazolam to suppress pharyngeal reflex is weaker than that of propofol, and the amount of sputum produced during anesthesia is increased. A muscle relaxant can be used during induction of anesthesia to suppress pharyngeal reflex, but the reflex becomes more pronounced when the muscle relaxant effect diminishes toward the awake phase. Second, remimazolam can maintain the intraoperative blood pressure at a higher level compared with propofol, which is one of the advantages of using remimazolam. Readers will also understand that intracranial pressure naturally increases when the blood pressure rises beyond autoregulation of cerebral blood flow, making it difficult for the surgeon to operate. Third, in one of our cases, the patient became agitated during the awake phase after antagonism with flumazenil. After immediately re-anesthetizing using propofol, the motor evoked potential (MEP) waveform temporarily disappeared. MEP gradually recovered, but there was a period when we could not monitor the MEP waveform, which was confusing for the surgeon and the MEP measurer. The precise mechanism for this response is unknown; however, we consider that remimazolam and propofol may have additively interfered with MEP. A previous study has reported that administration of midazolam or propofol moderately affected the MEP waveform [4], which suggests that it is important to verify the interaction between remimazolam and propofol.

We believe that remimazolam for awake craniotomy is theoretically correct and should be recommended; however, considering the concerns outlined above, it is controversial to state that it is beneficial for all cases during AC, and we think that further study is necessary. We suggest that JACR readers should assess whether or not remimazolam is appropriate for use for AC on a case-by-case basis.

## Data Availability

Not applicable

## Abbreviations

AC: Awake craniotomy
MEP: Motor evoked potential
